# Antibiotic resistance and serotype distribution of *Shigella* strains in Bangladesh over the period of 2014–2022: evidence from a nationwide hospital-based surveillance for cholera and other diarrheal diseases

**DOI:** 10.1128/spectrum.00739-24

**Published:** 2024-11-14

**Authors:** Mokibul Hassan Afrad, Md. Taufiqul Islam, Yasmin Ara Begum, Md. Saifullah, Faisal Ahmmed, Zahid Hasan Khan, Zakir Hossain Habib, Ahmed Nawsher Alam, Tahmina Shirin, Taufiqur Rahman Bhuiyan, Edward T. Ryan, Ashraful Islam Khan, Firdausi Qadri

**Affiliations:** 1 International Centre for Diarrheal Disease Research Bangladesh, Dhaka, Bangladesh; 2 Institute of Epidemiology, Disease Control and Research (IEDCR), Dhaka, Bangladesh; 3 Department of Medicine, Infectious Diseases Division, Massachusetts General Hospital, Boston, Massachusetts, USA; MultiCare Health System, Tacoma, Washington, USA

**Keywords:** diarrhea, *Shigella*, prevalence, antiobiotic resistance, nationwide surveillance, Bangladesh

## Abstract

The objective of this study was to assess the prevalence, antimicrobial resistance patterns, and risk factors linked to *Shigella* infections through a nationwide hospital-based diarrheal disease surveillance in Bangladesh. From May 2014 to May 2022, from a systematic sentinel surveillance of *Shigella* infections in over 10 hospitals across Bangladesh, stool specimens were collected from patients with acute watery diarrhea and tested for *Shigella* species by microbiological culture. The susceptibility to antibiotics was tested using the disk diffusion method. Structured questionnaires were used to collect participants’ socioeconomic status and clinical, sanitation, and food history. Out of 24,357 stool specimens, 1.8% were positive for *Shigella* species, with a higher prevalence among males (58%). Children in the 6–17 age group were found to be at the highest risk of *Shigella* infections. The most prevalent serotype was *Shigella flexneri* (79.5%), followed by *Shigella sonnei. S. flexneri* serotype 2a was the most common (63.3%) among all *Shigella* serotypes. Antibiotic susceptibility testing showed over 99% of isolates resistant or with intermediate susceptibility to one of the seven antibiotics tested. About 96% of *S. flexneri* and all *S. sonnei* isolates demonstrated resistance to at least one quinolone class of antibiotics, particularly ciprofloxacin or nalidixic acid. *S. sonnei* showed higher antibiotic resistance and multidrug resistance compared to *S. flexneri*. The high level of resistance to ciprofloxacin highlights the need for more prudent use of this antibiotic and improved hygiene and sanitation. The study emphasized the importance of regular monitoring of drug resistance to effectively manage *Shigella* infections. These findings may provide the epidemiological evidence for conducting future appropriate *Shigella* vaccine clinical trials in Bangladesh.

**IMPORTANCE:**

This nationwide study in Bangladesh assessed *Shigella* infections from 2014 to 2022 from clinical samples. *S. flexneri* was predominant, with concerning antibiotic resistance, notably to ciprofloxacin and nalidixic acid in over 96% of isolates. This emphasizes the urgency of prudent antibiotic use and improved hygiene. The findings provide crucial antimicrobial resistance patterns of *Shigella* species, highlighting the need for ongoing resistance monitoring and potentially informing future vaccine trials.

## INTRODUCTION

*Shigella* spp. are a major cause of acute watery diarrhea leading to complications particularly in low-income countries (LMIC )where healthcare facilities, water and sanitation systems, and treatment options are limited (1). In 2016, *Shigella* was estimated to cause 2.1 million deaths, accounting for around 13.2% of all diarrhea-related deaths (2). The Global Enteric Multicenter Study (GEMS) suggested that the burden of *Shigella* may be twice as high as previously estimated, ranking it as the most commonly detected pathogen (3).

The genus *Shigella* is composed of four species, including *Shigella flexneri*, *Shigella sonnei*, *Shigella dysenteriae*, and *Shigella boydii*, which differ based on the O antigen present on lipopolysaccharide walls (4). The four *Shigella* species and their different serotypes vary in their geographical distribution and epidemiological significance. Currently, the two most common species causing shigellosis are *S. flexneri* and *S. sonnei*, with *S. flexneri* being more prevalent in Asia and Africa and *S. sonnei* being more common in high-income countries (5, 6). However, the distribution of *S. sonnei* is increasing globally, including in LMICs) and is replacing *S. flexneri* as the predominant species (5–7).

The World Health Organization (WHO) advises that all instances of bloody diarrhea be treated immediately with an antimicrobial effective against *Shigella* to minimize the risk of complications, shorten the duration of illness, and prevent transmission to others (8). However, the primary challenge in treating *Shigella* is the emergence of multidrug-resistant strains, including growing resistance to third-generation cephalosporins, fluoroquinolones, and azithromycin (AZM) (9, 10). Fluoroquinolones such as ciprofloxacin (CIP) are currently the recommended first-line treatment for shigellosis, but in Asian countries like Bangladesh, the effectiveness of this antibiotic has been compromised by the emergence of resistant *Shigella* varieties (9, 11, 12).

Here, we present data from the nationwide hospital-based foodborne illness surveillance, where monitoring of *Shigella* antibiotic resistance has been ongoing since 2014. Additionally, we discuss the distribution of *Shigella* serotypes and antibiotic resistance pattern over a 9-year span in Bangladesh.

## MATERIALS AND METHODS

### Study site and population

**Fig 1:**
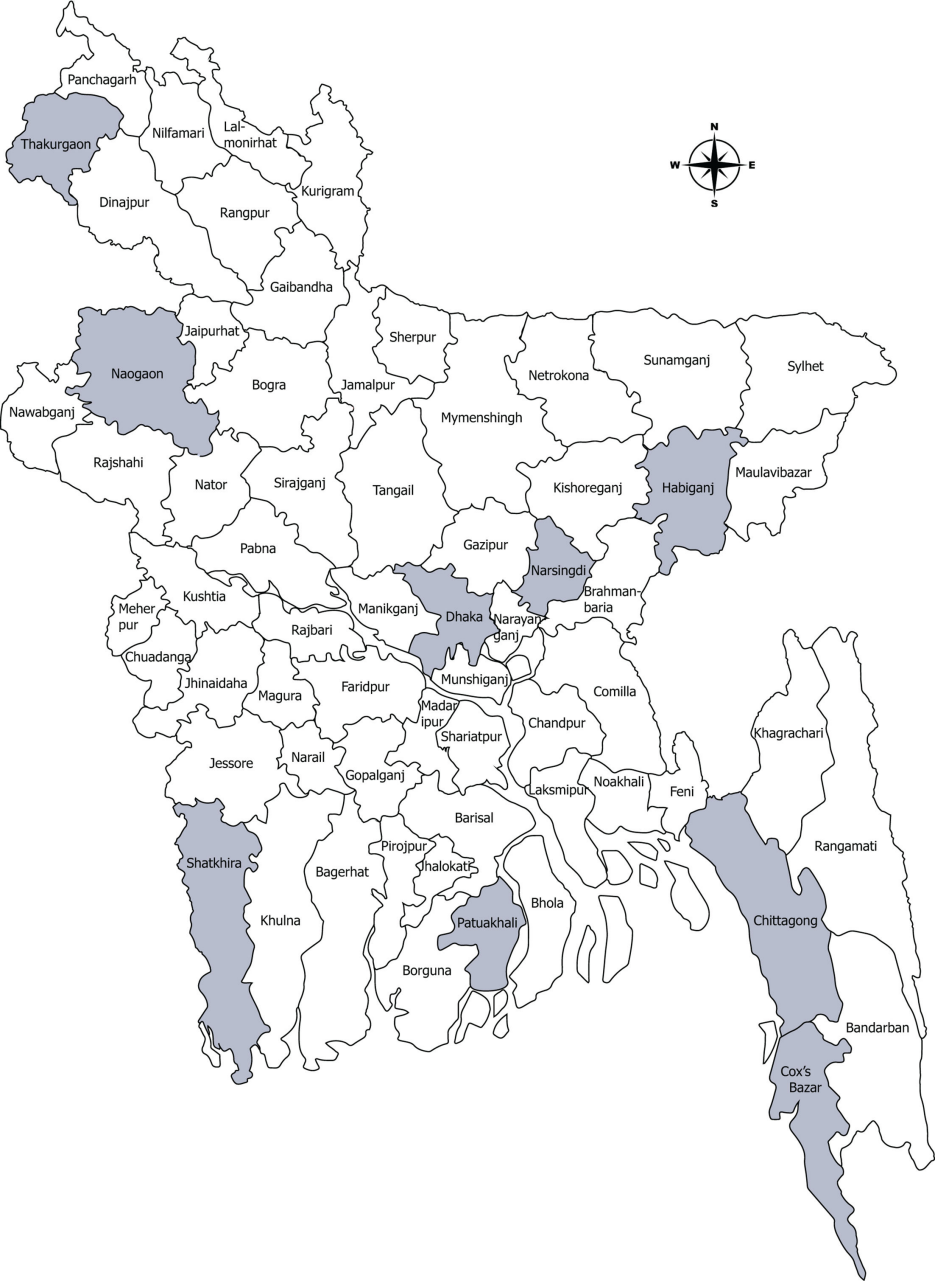
Map of nationwide study surveillance sites in Bangladesh, May 2014–May 2022. The map was created by ArcGIS Pro version 3.2.

In May 2014, the International Centre for Diarrhoeal Disease Research, Bangladesh (icddr,b), and the Institute of Epidemiology, Disease Control and Research (IEDCR), Bangladesh, collaboratively started the enteric food-borne illness surveillance in 10 hospitals in eight different districts in Bangladesh (Fig. 1). However, there was a gap in funding which resulted in the interruption of the surveillance from January to May of 2016. The 10 sites were selected based on reports of acute watery diarrhea and previous surveillance studies, from the Directorate General of Health Services and other sources (13, 14). In this study, participants were enrolled from May 2014 to May 2022.

### Surveillance

The surveillance methods utilized in this study have been previously described in Khan et al. (15). The procedure has been summarized below for the reader’s convenience.

Surveillance covered all age groups, but distinct case definitions were applied for those under 2 months and those 2 months or older: (i) for infants under 2 months, changed stool habit from usual pattern in terms of frequency (more than the usual number of purgings) or nature of stool (more water than fecal matter); (ii) for individuals aged 2 months or older, diarrhea was defined as any patient presenting at the hospital with three or more loose or liquid stools within 24 h, or with three or fewer loose/liquid stools leading to dehydration within the last 24 h.

At each surveillance site, a team including a physician, nurse, medical technologist, and trained field attendant was established. At each site, a daily list of diarrhea patients was prepared (both inpatient and outpatient). Four patients meeting the case definition, without severe comorbidities (e.g., severe acute respiratory illness, acute cardiovascular symptoms, or severe acute neurological disorder), were enrolled each day from Saturday to Wednesday. Two patients under 5 years old and two patients aged 5 years or older were enrolled each day. If the target number was not met in one age group, patients from the other group were included to meet the target of four patients. Upon receiving consent, the physician collected sociodemographic characteristics, such as age, gender, profession, diet history, medical history, sanitation, and hygiene information, and a stool sample for testing. The specimens were collected in Cary-Blair transport media and buffered glycerol saline (BGS) media and transported within 15 days to the laboratories in Dhaka at the icddr,b, and IEDCR, following cold-chain procedures (2°C–8°C), for immediate laboratory processing.

### Laboratory procedure

The conventional microbiological culture was carried out from both Carry-Blair and BGS independently by streaking directly on *Salmonella Shigella* (SS) agar (Becton Dickinson, France) and incubated at 37°C for 16–22 h. The resultant *Shigella*-like colonies were picked onto Kligler iron agar, motility indole urea, and Simmon citrate agar (Becton Dickinson) and then analyzed further through biochemical tests to identify and isolate any potential *Shigella* colonies. The serotyping of *Shigella* isolates was carried out using *Shigella* Antisera (Denka Seiken, Tokyo, Japan) and monoclonal antibody reagents (MASF IV-1 and MASF IV-2; Reagensia AB, Stockholm, Sweden).

### Antimicrobial susceptibility testing

Susceptibility to antimicrobial agents, including ampicillin (AMP, 10 µg), AZM (15 µg), CIP (5 µg), ceftriaxone (CRO, 30 µg), mecillinam (MEL, 10 µg), nalidixic acid (NA, 30 µg), and sulfomethoxazole-trimethoprim (SXT, 1.25/23.75 µg) (Oxoid, Basingstoke, United Kingdom), was determined by the disk diffusion method, which was recommended by the Clinical and Laboratory Standards Institute (16). For *S. flexneri*, the susceptibility of every fifth positive isolate was tested (*n* = 185), while for *S. sonnei* (*n* = 68), all of the isolates were tested. *Escherichia coli* American Type Culture Collection 25922 strain was used as the susceptible control strain.

### Statistical analysis

The data were entered into Microsoft Structured Query Language server for analysis. The demographics, clinical characteristics, and behavioral components were summarized using proportions for categorical variables and median with interquartile range for continuous variables. Bivariate analysis using Pearson’s chi-squared test was performed for the primary analysis to determine any statistical differences, with a significance level of *P* ≤ 0.05. Logistic regression was used to identify the predictors of enteric diseases that fit in the model with a significance level of *P* < 0.05 from the chi-squared test, and the results were expressed as crude and adjusted odds ratios.

## RESULTS

From May 2014 to May 2022, a total of 31,517 patients were enrolled in the surveillance (Table 1), with 55.7% being male. Over 50% of the patients with diarrhea enrolled were under five years old, 30.3% were between 18 and 45 years old, and 7% were between 46 and 60 years old (Table 2). About 20% of the enrolled patients were housewives and 6.7% were service holders (e.g. teachers, bankers, and public servants). Most patients reported some level (61.5%) or severe (12.7%) of dehydration. Other reported symptoms included vomiting (63.4%), fever (53.5%), and abdominal cramps (50.7%). 25% of the patients had consumed food from roadside vendors, 82.6% had drunk untreated water, and 20.8% had neighbors with diarrhea in the week prior to their illness.

**TABLE 1:** Site-wise distribution of diarrhea samples and *Shigella*-positive organisms from May 2014 to May 2022

Division	Surveillance sites	Participants enrolled	Culture performed	*Shigella* positive, *n* (%)	
				Site-wise	Division-wise
Dhaka	Narsingdi	1,745	1,737	32 (1.8)	97 (2.3)
	DMCH,^*a*^ South Dhaka	1,960	1,453	30 (2.1)	
	UAMC&H,^*b*^ North Dhaka	1,539	1,036	35 (3.4)	
Chattogram	Cox’s Bazar	4,385	3,298	71 (2.2)	127 (2)
	BITID^*c*^	3,911	2,852	56 (2)	
Sylhet	Habiganj	4,816	3,564	71 (2)	71 (2)
Rajshahi	Naogaon	3,570	2,601	50 (1.9)	50 (1.9)
Barisal	Patuakhali	3,946	2,930	52 (1.8)	52 (1.8)
Rangpur	Thakurgaon	2,439	2,417	7 (0.3)	7 (0.3)
Khulna	Shatkhira	3,206	2,469	41 (1.7)	41 (1.7)
Total		31,517	24,357	445 (1.8)	445 (1.8)

^*a*^ DMCH, Dhaka Medical College Hospital.

^*b*^ UAMC&H, Uttara Adhunik Medical College and Hospital.

^*c*^ BITID, Bangladesh Institute of Tropical and Infection Diseases.

**TABLE 2:** Factors associated with *Shigella*

Characteristics	*N* = 24,357^*a*^	Negative, *n* = 23,912^*a*^	Positive, *n* = 445^*a*^	*P* value^*b*^
Age group (years)				**<0.001**
0–5	12,777 (52.5)	12,588 (98.5)	189 (1.5)	
6–17	986 (4.0)	941 (95.4)	45 (4.6)	
18–45	7,372 (30.3)	7,250 (98.3)	122 (1.7)	
46–60	1,670 (6.9)	1,637 (98.0)	33 (2.0)	
≥61	1,552 (6.4)	1,496 (96.4)	56 (3.6)	
Age in years	3 (0, 35)	3 (0, 35)	14 (2, 40)	**<0.001**
Gender				0.300
Female	10,797 (44.3)	10,611 (98.3)	186 (1.7)	
Male	13,560 (55.7)	13,301 (98.1)	259 (1.9)	
Occupation				**<0.001**
Service holder	1,635 (6.7)	1,611 (98.5)	24 (1.5)	
Housewife	4,772 (19.6)	4,678 (98.0)	94 (2.0)	
Agriculture worker	830 (3.4)	810 (97.6)	20 (2.4)	
Businessman	979 (4.0)	963 (98.4)	16 (1.6)	
Labor/worker/driver	800 (3.3)	782 (97.8)	18 (2.2)	
Student and unemployed	1,986 (8.2)	1,924 (96.9)	62 (3.1)	
Child (up to 10 years)	13,149 (54.0)	12,941 (98.4)	208 (1.6)	
Others	206 (0.8)	203 (98.5)	3 (1.5)	
Duration of diarrhea (days)				0.009
<3	12,708 (52.2)	12,444 (97.9)	264 (2.1)	
3–7	11,463 (47.1)	11,285 (98.4)	178 (1.6)	
≥8	186 (0.8)	183 (98.4)	3 (1.6)	
Number of purging in last 24 h				0.400
≤10, times	9,248 (38.0)	9,071 (98.1)	177 (1.9)	
>10, times	15,109 (62.0)	14,841 (98.2)	268 (1.8)	
Nature of stool				**<0.001**
Loose watery	13,159 (54.0)	12,918 (98.2)	241 (1.8)	
Rice watery	10,101 (41.5)	9,945 (98.5)	156 (1.5)	
Bloody	121 (0.5)	91 (75.2)	30 (24.8)	
Formed	976 (4.0)	958 (98.2)	18 (1.8)	
Vomiting				0.021
No	8,906 (36.6)	8,720 (97.9)	186 (2.1)	
Yes	15,451 (63.4)	15,192 (98.3)	259 (1.7)	
Dehydration				**<0.001**
No	6,277 (25.8)	6,168 (98.3)	109 (1.7)	
Some	14,991 (61.5)	14,679 (97.9)	312 (2.1)	
Severe	3,089 (12.7)	3,065 (99.2)	24 (0.8)	
Abdominal cramp				**<0.001**
No	12,020 (49.3)	11,850 (98.6)	170 (1.4)	
Yes	12,337 (50.7)	12,062 (97.8)	275 (2.2)	
Fever				**<0.001**
No	11,318 (46.5)	11,147 (98.5)	171 (1.5)	
Yes	13,039 (53.5)	12,765 (97.9)	274 (2.1)	
Tap water				0.023
No	18,155 (74.5)	17,844 (98.3)	311 (1.7)	
Yes	6,202 (25.5)	6,068 (97.8)	134 (2.2)	
Tube well				0.026
No	4,134 (17.0)	4,041 (97.8)	93 (2.2)	
Yes	20,223 (83.0)	19,871 (98.3)	352 (1.7)	
Bottled water				0.300
No	21,428 (88.0)	21,030 (98.1)	398 (1.9)	
Yes	2,929 (12.0)	2,882 (98.4)	47 (1.6)	
Water treated by boiled/filtered/chemical				0.800
No	20,128 (82.6)	19,762 (98.2)	366 (1.8)	
Yes	4,229 (17.4)	4,150 (98.1)	79 (1.9)	
Take food from roadside				0.200
No	18,209 (74.8)	17,888 (98.2)	321 (1.8)	
Yes	6,148 (25.2)	6,024 (98.0)	124 (2.0)	
Take food from large gatherings				0.200
No	21,203 (87.1)	20,825 (98.2)	378 (1.8)	
Yes	3,154 (12.9)	3,087 (97.9)	67 (2.1)	
Any one of neighbors has the same disease				0.600
No	19,291 (79.2)	18,934 (98.1)	357 (1.9)	
Yes	5,066 (20.8)	4,978 (98.3)	88 (1.7)	

^*a*^ *n* (%), median (interquartile range).

^*b*^ Pearson’s chi-squared test, Wilcoxon’s rank-sum test, Fisher’s exact test. Statistically significant values (*P* < 0.05) are highlighted in bold text.

Out of the 31,517 patients enrolled, 24,357 stool specimens were available for microbiological culture, and 1.8% (445/24,357) of these tested positive for *Shigella* spp (Table 1). The majority of the positive cases were under 5 years old and 27% were 18–45 years old, 58% were male (Table 2). In these cases, majority of the cases reported some (70%) or severe (5.4%) dehydration, 58% had vomiting, 62% had abdominal cramps, and 60% reported purging rates of more than 10 times in the last 24 h. About, 28% of those positive had consumed food from street vendors, 82% had drunk untreated water, and 19.8% had neighbors with diarrhea in the week before their illness.

We found that the risk of *Shigella* infection was lower among patients with diarrhea who were younger than 6 years old compared to other age groups (Table 3). The risk was 2.4 times higher in those between 6 and 17 years old (95% CI: 1.57–3.78) and 1.96 times higher in those over 61 years old (95% CI: 1.01–3.8) compared to those under 6. Additionally, patients infected with *Shigella* had an 11.41 times higher risk (95% CI: 8.21–15.85) of having blood in their stool.

**TABLE 3:** Clinical data and risk factors of stool culture-positive *Shigella* [using generalized linear mixed-effect models (random: surveillance sites)]*^a^^,b^*

Characteristics	Labels	cOR (95% CI)	*P* value^*c*^	aOR (95% CI)	*P* value
Age (years)	<6 (ref)	1.00			
	6–17	2.94 (2.09–4.13)	**<0.001**	2.44 (1.57–3.78)	**<0.001**
	18–45	1.02 (0.8–1.3)	0.879	0.98 (0.5–1.9)	0.95
	46–60	1.23 (0.84–1.8)	0.282	1.14 (0.56–2.35)	0.714
	≥61	2.29 (1.67–3.14)	**<0.001**	1.96 (1.01–3.8)	**0.047**
Occupation	Service holder (ref)	1.00			
	Housewife	1.43 (0.9–2.27)	0.126	1.39 (0.88–2.17)	0.155
	Agriculture worker	1.86 (1.0–3.43)	**0.048**	1.62 (0.89–2.96)	0.115
	Businessman	1.16 (0.61–2.21)	0.649	1.15 (0.61–2.14)	0.664
	Labor/worker/driver	1.63 (0.88–3.02)	0.124	1.64 (0.9–2.99)	0.103
	Student and unemployed	2.27 (1.4– 3.68)	**0.001**	1.5 (0.9–2.5)	0.119
	Child (up to 10 years)	1.28 (0.82–2.01)	0.277	1.47 (0.72–3.01)	0.292
	Others	1.01 (0.29–3.47)	0.986	0.83 (0.25–2.7)	0.753
Duration of diarrhea (days)	<3 (ref)	1.00			
	3–7	0.84 (0.69–1.04)	0.105	0.79 (0.64–0.97)	**0.022**
	≥8	0.81 (0.25–2.56)	0.715	0.63 (0.21–1.86)	0.399
Nature of stool	Loose watery (ref)	1.00			
	Rice watery stool	0.83 (0.64–1.06)	0.131	0.85 (0.66–1.08)	0.184
	Bloody	17.08 (11.02–26.48)	**<0.001**	11.41 (8.21–15.85)	**<0.001**
	Formed	0.93 (0.56–1.55)	0.793	0.88 (0.54–1.43)	0.601
Vomiting	Yes	0.76 (0.61–0.93)	**0.010**	0.72 (0.59–0.88)	**0.002**
Dehydration	None	1.00			
	Some	1.2 (0.94–1.52)	0.148	1.13 (0.9–1.43)	0.301
	Severe	0.56 (0.35–0.91)	**0.018**	0.55 (0.34–0.89)	**0.014**
Abdominal cramp	Yes	1.38 (1.11–1.7)	**0.003**	1.18 (0.93–1.51)	0.176
Fever	Yes	1.44 (1.17–1.78)	**0.001**	1.42 (1.16–1.73)	**0.001**
Tap water	Yes	1.03 (0.8–1.32)	0.818	1.09 (0.82–1.45)	0.562
Tube well	Yes	0.91 (0.67–1.24)	0.558	0.95 (0.69–1.31)	0.751

^*a*^ Note: all no exposures as reference.

^*b*^ aOR, adjusted odds ratio; cOR, crude odds ratio.

^*c*^ P values ≤0.05 are presented in bold.

The isolation rate of *Shigella* varied from 0.3% to 3.4% (Table 1), and phenotypic identification revealed that 79.5% were *S. flexneri*, 18.8% were *S. sonnei*, 1.2% were *S. boydii*, and 0.5% were *S. dysenteriae* (Table 4). Throughout the study, *S. flexneri* remained the most prevalent strain. The isolation rate of *S. sonnei* increased over the years, with the highest rate seen in 2021 (33.3%). The most prevalent *S. flexneri* serotype was 2 a (79.7%) followed by 3 a (10.8%). Six (1.8%) *S. flexneri* isolates were not able to be typed. For *S. sonnei*, phase-I serotype was the most common from 2014 to 2019.

**TABLE 4:** *Shigella* serotypes in Bangladesh from May 2014 to May 2022

*Shigella* spp.	2014 *n* (%)	2015 *n* (%)	2016^*b*^ *n* (%)	2017 *n* (%)	2018 *n* (%)	2019 *n* (%)	2020 *n* (%)	2021 *n* (%)	2022 *n* (%)	Grand total *N* (%)
*S. flexneri* ***^a^***	26 (86.7)	46 (100)	26 (86.7)	62 (75.6)	45 (84.9)	37 (72.5)	35 (76.1)	34 (66.7)	23 (74.2)	334 (79.5)
6				1 (1.6)						1 (0.3)
1a				1 (1.6)						1 (0.3)
1b					3 (6.7)					3 (0.9)
1c		1 (2.2)	1 (3.8)	3 (4.8)		1 (2.7)				6 (1.8)
2a	15 (57.7)	37 (80.4)	19 (73.1)	45 (72.6)	36 (80.0)	34 (91.9)	34 (97.1)	28 (82.4)	18 (78.3)	266 (79.6)
3a	9 (34.6)	3 (6.5)	3 (11.5)	10 (16.1)	4 (8.9)		1 (2.9)	4 (11.8)	2 (8.7)	36 (10.8)
4a		2 (4.3)	3 (11.5)	1 (1.6)	1 (2.2)	1 (2.7)				8 (2.4)
X				1 (1.6)				1 (2.9)	3 (13.0)	5 (1.5)
Y	1 (3.8)					1 (2.7)				2 (0.6)
Untypeable	1 (3.8)	3 (6.5)			1 (2.2)			1 (2.9)		6 (1.8)
*S. sonnei*	3 (10.0)		3 (10.0)	20 (24.4)	5 (9.4)	13 (25.5)	11 (23.9)	17 (33.3)	7 (22.6)	79 (18.8)
Phase I	3 (100.0)		2 (66.7)	11 (55.0)	3 (60.0)	7 (53.8)	4 (36.4)	2 (11.8)	1 (14.3)	33 (41.8)
Phase II			1 (33.3)	9 (45.0)	2 (40.0)	6 (46.2)	7 (63.6)	15 (88.2)	6 (85.7)	46 (58.2)
*S. dysenteriae*	1 (3.3)				1 (1.9)					2 (0.5)
*S. boydii*			1 (3.3)		2 (3.8)	1 (2.0)			1 (3.2)	5 (1.2)
Total	30 (100.0)	46 (100.0)	30 (100.0)	82 (100.0)	53 (100.0)	51 (100.0)	46 (100.0)	51 (100.0)	31 (100.0)	420 (100.0)

^*a*^ The proportion of *S. flexneri* and *S. sonnei* subspecies was calculated based on the total number of positive isolates for each respective species.

^*b*^ In 2016, samples were collected from June to December only.

### *Shigella* AMR in Bangladesh

Out of the 253 *S*. *flexneri* and *S. sonnei* isolates tested for antibiotic sensitivity, 98.8% (250 of 253) were either resistant or had intermediate susceptibility to at least one of the seven antibiotics tested (Table 5). Of the 185 *S*. *flexneri* isolates, 96% were found to be resistant to at least one of the quinolone class of antibiotics, such as CIP or NA.

**TABLE 5:** Antimicrobial resistance profiles of *Shigella* isolates in Bangladesh from May 2014 to May 2022

	*S. flexneri* (*n* = 185)			*S. sonnei* (*n* = 68)		
Antimicrobial agents	S	I	R	S	I	R
Ampicillin	100 (54.1%)	0	85 (46%)	23 (33.8%)	0	45 (66.2%)
Azithromycin	141 (76.3%)	4 (2.2%)	40 (21.7%)	10 (14.7%)	1 (1.5%)	57 (83.8%)
Ciprofloxacin	29 (15.7%)	13 (7.1%)	143 (77.3%)	0	1 (1.5%)	67 (98.5%)
Ceftriaxone	179 (96.8%)	2 (1.1%)	4 (2.2%)	29 (42.6%)	25 (36.8%)	14 (20.6%)
Mecillinam	172 (93%)	6 (3.3%)	7 (3.8%)	68 (100%)	0	0
Nalidixic acid	11 (6%)	2 (1.1%)	172 (93%)	0	0	68 (100%)
Trimethoprim/sulfamethoxazole	100 (54.1%)	0	85 (46%)	9 (13.2%)	9 (13.2%)	50 (73.5%)

**TABLE 6:** Multidrug resistance profiles of *Shigella* isolates in Bangladesh from May 2014 to May 2022^*a*^

Antibiotic resistance	Number of isolates (%)		MDR profile	*S. flexneri* *n* (%)	*S. sonnei* *n* (%)	Total
	*S. flexneri*, *n* = 182	*S. sonnei*, *n* = 68				
≥1 CLSI class	182	68	AMP-CIP-NA	24 (96)	1 (4)	25
≥2 CLSI class	162	66	AMP-AZM-CIP-CRO-NA-SXT	1 (3.6)	27 (96.4)	28
≥3 CLSI class	112	60	CIP-NA-SXT	22 (91.7)	2 (8.3)	24
≥4 CLSI class	58	50	AMP-CIP-NA-SXT	17 (100.0)	0	17
≥5 CLSI class	22	43	AMP-AZM-CIP-NA-SXT	15 (88.2)	2 (11.8)	17
			AMP-AZM-CRO-NA-SXT	2 (12.5)	14 (87.5)	16
			AZM-CIP-NA-SXT	8 (66.7)	4 (33.3)	12
			AMP-AZM-CIP-NA	6 (100.0)	0	6
			AZM-NA-SXT	1 (14.3)	6 (85.7)	7
			AZM-CIP-NA	5 (83.3)	1 (16.7)	6
			AZM-CIP-MEL-NA	3 (100.0)	0	3
			AMP-AZM-CIP-MEL-NA	3 (100.0)	0	3
			AMP-NA-SXT	1 (50.0)	1 (50.0)	2
			AMP-MEL-NA	1 (100.0)	0	1
			AMP-CIP-MEL-NA	1 (100.0)	0	1
			AMP-CIP-CRO-NA-SXT	1 (100.0)	0	1
			AMP-AZM-NA-SXT	1 (33.3)	2 (66.7)	3
			AMP-AZM-CRO-NA	0	1 (100.0)	1
			AMP-AZM-CIP-CRO-NA	0	1 (100%)	1

^*a*^ CLSI, Clinical and Laboratory Standards Institute; MDR, multidrug resistant.

Of the *S. flexneri* isolates, 94% were found to have either intermediate resistance or resistance to NA, followed by CIP (84.4%), SXT (46%), ampicillin (46%), AZM (23.8%), MEL (7.1%), and CRO (3.3%). Meanwhile, *S. sonnei* isolates showed higher resistance rates to a variety of antibiotics, including 100% resistance to NA, followed by 98.5% to CIP, 83.8% to AZM, 73.5% to SXT, 66.2% to AMP, and 20.6% to CRO.

Out of the 253 *Shigella* isolates tested for antibiotic resistance, 29 different resistance patterns were identified (Table 5). Out of these, 69% (20 out of 29) were multidrug resistant (MDR), meaning they were resistant to at least three classes of antibiotics. Of the total 253 isolates, 175 (69%) were MDR, with 112 out of 182 (61.5%) *S*. *flexneri* isolates and 60 out of 68 (88.2%) *S*. *sonnei* isolates being MDR. The study found that *S. sonnei* had a higher frequency of MDR compared to *S. flexneri* isolates.

## DISCUSSION

This study presents data on the prevalence and types of *Shigella* spp. isolated in patients with acute diarrhea and the clinical characteristics and risk factors associated with *Shigella* infections in Bangladesh.

We found that *Shigella* is prevalent throughout the country, and its incidence has remained stable over the course of the study, with a range from from 0.3% to 3.4% (Table 1). Division-wise (administrative area), except Rangpur Division, all other administrative divisions exhibited a similar *Shigella* detection rate ranging from 1.7% to 2.3%, whereas Rangpur Division recorded the lowest detection rate at 0.3% (Table 1).

*Shigella* infection was seen in people of all ages, but children under 5 years old had the most cases. The highest positive detection rate was observed in children aged 6–17 years. Males were more likely to be infected with *Shigella*, with 58% of the cases being male, which aligns with previous studies by Khan et al. and Taneja (17, 18).

The study found that *S. flexneri* was the most prevalent species of the *Shigella* genus, accounting for 80% of cases, which is in line with other studies conducted in Bangladesh and developing countries in Africa and Asia (19). However, there was a significant increase in the prevalence of *S. sonnei* cases, rising from 10% in 2014 to 24.4% in 2017. Recent evidence suggests that *S. sonnei* is emerging as the dominant cause of shigellosis in countries undergoing economic transition (20), and this study showed a similar trend.

Recently, *S. sonnei* has become more prevalent in certain regions of Bangladesh, such as Shatkhira district, where it was found in 78.3% of cases. Over the years, *S. sonnei* has become more frequent in Bangladesh, from 12% in 2004 to 25% in 2011 in urban areas (14), and from 35% in 2010 to 41% in 2012 in rural areas (7, 21). Such increase has also been documented in other Asian countries such as India (22), Pakistan (23), Vietnam (24), and Thailand (25). The GEMS has recognized this trend and recommended the development of a quadrivalent vaccine to protect against *S. sonnei* and *S. flexneri* (serotypes 2a, 3a, and 6) in endemic regions (19). Research has linked this increase in *S. sonnei* to economic growth (20). As Bangladesh continues to develop economically and improve its sanitation, *S. sonnei* may become a more significant public health concern in the future.

The study found that *S. flexneri* 2a was still the most prevalent serotype in Bangladesh, consistent with previous research. Seven subtypes of *S. flexneri* were identified; serotype 2a was the most widespread subtype, accounting for 63% of *Shigella* cases. Additionally, atypical serotypes such as 1a, 1b, 1c, 4a, and 6 were also detected.

Antimicrobial resistance has been the key driver of the evolution of *Shigella* species, especially in the LMICs. This has been attributed to factors such as over-prescription and easy access to antibiotics (26, 27). The study found that 98.8% of the tested *Shigella* isolates in Bangladesh showed resistance or intermediate susceptibility to at least one of the seven antibiotics, and 41.8% had MDR profiles (Tables 5 and 6). There were differences in the antibiotic resistance profile between *S. flexneri* and *S. sonnei*, with the latter having a higher MDR level of 92.6%. Sixty-six percent of *S. sonnei* strains were resistant to commonly used antibiotics such as AMP, AZM, or CIP, while the dominant resistance profile among *S. flexneri* was AMP-CIP-NA-SXT (81.8%). A previous study in Bangladesh documented that 95% of *S. sonnei* and 66% of *S. flexneri* had MDR profiles, and a small number of isolates were found to be extensively drug-resistant (28).

The study found differences in the AMR profiles between *S. flexneri* and *S. sonnei*. According to the WHO, CIP is recommended as the first-line treatment for shigellosis (27), a high percentage of both *S. flexneri* and *S. sonnei* strains showed resistance or intermediate resistance to CIP, with resistance rates of 77% and 98%, respectively ()(Table 5). Among previous studies conducted in Bangladesh, Azmi et al. also reported a gradual increase in resistance of CIP from 0% in 2004 to 44% in 2010 (29). Ud-Din et al. also showed evidence of a dramatic change of resistance of *S. sonnei* to CIP from 10% in 2007 to 70% in 2011 (7). Similar trends have been observed in other South Asian countries like India, Nepal, and China (30, 31). The *S. sonnei* strains also showed a high resistance (66.2%) to AMP, while *S. flexneri* showed 46% resistance to AMP.

Previous studies from Southeast Asia reported the resistance of *Shigella* spp. to cephalosporins at 2.0%–5.2% (22, 30). In this study, we found a higher resistance to cephalosporins, with 20.6% of *S. sonnei* isolates showing resistance and 36.8% showing intermediate resistance to CRO, while *S. flexneri* showed 2% resistance. A large multicenter study in eight Asian countries from 2001 to 2004 also reported an increase in CRO resistance (5%) among *Shigella* isolates (30). These findings suggest that the most suitable antibiotic for treating *Shigella* infections may vary between *S. sonnei* and *S. flexneri*, with the former being more challenging to treat. The study found that resistance to SXT and NA was high from 2014 to 2017, while resistance to MEL was lower (2.8%).

The strengths of our study included an unbiased sampling approach that considered factors such as age, sex, nutrition status, disease severity, and socioeconomic context and samples from eight different geographical locations over a 9-year period. However, there are also several limitations. Due to funding constraints, the study was only conducted at 10 sentinel sites, which may not accurately reflect the full diversity of *Shigella* epidemiology and burden within the country. Moreover, the study was halted due to funding gap from January to May of 2016. A higher density surveillance network may provide more detailed insights. Additionally, the study only used conventional culture methods, not PCR, for confirming *Shigella* cases, which may have limited sensitivity (3), especially since around 46% of participants reported taking at least one dose of antibiotics for their current illness before enrollment. The use of quantitative PCR has revealed a higher burden of Shigella-attributable diarrhea in low-resource settings than previously recognized with culture-based diagnostics (32, 33). The study also did not perform genetic typing, which could have added valuable insight into the mechanisms of antibiotic resistance. The results of antibiotic resistance testing were only qualitative, not quantitative, and did not determine resistance levels that may be considered resistance. Lastly, there was no follow-up with patients after treatment or discharge, resulting in a lack of data on clinical outcomes and mortality.

In conclusion, our research indicated a noticeable rise in the prevalence of *S. sonnei* as the second most common *Shigella* species over the years. Despite having a high level of sensitivity to MEL, the *Shigella* strains in the study demonstrated resistance to antibiotics like AMP, AZM, NA, and SXT. The widespread resistance to fluoroquinolones and third-generation cephalosporins limits the available treatment options for shigellosis. This rising resistance to first-line antimicrobials emphasizes the need for new preventive and therapeutic measures. There are vaccines and alternative treatments in development, such as a live attenuated oral vaccine (WRSS1) against *S. sonnei* that has been found to be safe and effective in Bangladesh (34). Finally, ongoing monitoring of antibiotic drug susceptibility and determination of serotypes are necessary to comprehend the spread of *Shigella*.

The increasing resistance to fluoroquinolones and macrolides emphasizes the urgency for the development of a vaccine to prevent against *Shigella* infections. In order to plan for vaccine trials effectively and gain insights into the impact of *Shigella* infections in areas with limited resources, it is essential to have comprehensive data on *Shigella* infection epidemiology. Such surveillance is extremely important for preparation of preventive and vaccination efforts in Bangladesh and globally.

## Supplementary Material

Reviewer comments

## SUPPLEMENTAL MATERIAL

OPEN PEER REVIEW reviewer-comments.pdf An accounting of the reviewer comments and feedback.

ASM does not own the copyrights to Supplemental Material that may be linked to, or accessed through, an article. The authors have granted ASM a non-exclusive, world-wide license to publish the Supplemental Material files. Please contact the corresponding author directly for reuse.

## References

[1] GBD 2016 Diarrhoeal Disease Collaborators. 2018. Estimates of the global, regional, and national morbidity, mortality, and aetiologies of diarrhoea in 195 countries: a systematic analysis for the Global Burden of Disease Study 2016. Lancet Infect Dis 18:1211–1228. doi:10.1016/S1473-3099(18)30362-1 30243583 PMC6202444

[2] Khalil IA, Troeger C, Blacker BF, Rao PC, Brown A, Atherly DE, Brewer TG, Engmann CM, Houpt ER, Kang G, et al.. 2018. Morbidity and mortality due to Shigella and enterotoxigenic Escherichia coli diarrhoea: the global burden of disease study 1990-2016. Lancet Infect Dis 18:1229–1240. doi:10.1016/S1473-3099(18)30475-4 30266330 PMC6202441

[3] Liu J, Platts-Mills JA, Juma J, Kabir F, Nkeze J, Okoi C, Operario DJ, Uddin J, Ahmed S, Alonso PL, et al.. 2016. Use of quantitative molecular diagnostic methods to identify causes of diarrhoea in children: a reanalysis of the GEMS case-control study. Lancet 388:1291–1301. doi:10.1016/S0140-6736(16)31529-X 27673470 PMC5471845

[4] The HC, Thanh DP, Holt KE, Thomson NR, Baker S. 2016. The genomic signatures of Shigella evolution, adaptation and geographical spread. Nat Rev Microbiol 14:235–250. doi:10.1038/nrmicro.2016.10 26923111

[5] Holt KE, Baker S, Weill F-X, Holmes EC, Kitchen A, Yu J, Sangal V, Brown DJ, Coia JE, Kim DW, Choi SY, Kim SH, da Silveira WD, Pickard DJ, Farrar JJ, Parkhill J, Dougan G, Thomson NR. 2012. Shigella sonnei genome sequencing and phylogenetic analysis indicate recent global dissemination from Europe. Nat Genet 44:1056–1059. doi:10.1038/ng.2369 22863732 PMC3442231

[6] Qiu S, Xu X, Yang C, Wang J, Liang B, Li P, Li H, Yi S, Liu H, Cui X, Wu Z, Xie J, Jia L, Wang L, Hao R, Jin H, Wang Y, Sun Y, Song H. 2015. Shift in serotype distribution of Shigella species in China, 2003-2013. Clin Microbiol Infect 21:252. doi:10.1016/j.cmi.2014.10.019 25658535

[7] Ud-Din A, Wahid SUH, Latif HA, Shahnaij M, Akter M, Azmi IJ, Hasan TN, Ahmed D, Hossain MA, Faruque ASG, Faruque SM, Talukder KA. 2013. Changing trends in the prevalence of Shigella species: emergence of multi-drug resistant Shigella sonnei biotype g in Bangladesh. PLoS ONE 8:e82601. doi:10.1371/journal.pone.0082601 24367527 PMC3867351

[8] Williams PCM, Berkley JA. 2018. Guidelines for the treatment of dysentery (shigellosis): a systematic review of the evidence. Paediatr Int Child Health 38:S50–S65. doi:10.1080/20469047.2017.1409454 29790845 PMC6021764

[9] Bhattacharya D, Bhattacharya H, Sayi DS, Bharadwaj AP, Singhania M, Sugunan AP, Roy S. 2015. Changing patterns and widening of antibiotic resistance in Shigella spp. over a decade (2000-2011), Andaman Islands, India. Epidemiol Infect 143:470–477. doi:10.1017/S0950268814000958 24763083 PMC9507051

[10] Rahman M, Shoma S, Rashid H, El Arifeen S, Baqui AH, Siddique AK, Nair GB, Sack DA. 2007. Increasing spectrum in antimicrobial resistance of Shigella isolates in Bangladesh: resistance to azithromycin and ceftriaxone and decreased susceptibility to ciprofloxacin. J Health Popul Nutr 25:158–167. 17985817 PMC2753991

[11] Talukder KA, Khajanchi BK, Islam MA, Dutta DK, Islam Z, Safa A, Khan GY, Alam K, Hossain MA, Malla S, Niyogi SK, Rahman M, Watanabe H, Nair GB, Sack DA. 2004. Genetic relatedness of ciprofloxacin-resistant Shigella dysenteriae type 1 strains isolated in south Asia. J Antimicrob Chemother 54:730–734. doi:10.1093/jac/dkh425 15347639

[12] Talukder KA, Khajanchi BK, Islam MA, Islam Z, Dutta DK, Rahman M, Watanabe H, Nair GB, Sack DA. 2006. Fluoroquinolone resistance linked to both gyrA and parC mutations in the quinolone resistance-determining region of Shigella dysenteriae type 1. Curr Microbiol 52:108–111. doi:10.1007/s00284-005-0140-9 16450072

[13] Sack RB, Siddique AK, Longini IM Jr, Nizam A, Yunus M, Islam MS, Morris JG Jr, Ali A, Huq A, Nair GB, Qadri F, Faruque SM, Sack DA, Colwell RR. 2003. A 4-year study of the epidemiology of Vibrio cholerae in four rural areas of Bangladesh. J Infect Dis 187:96–101. doi:10.1086/345865 12508151

[14] Talukder KA, Islam Z, Dutta DK, Islam MA, Khajanchi BK, Azmi IJ, Iqbal MS, Hossain MA, Faruque ASG, Nair GB, Sack DA. 2006. Antibiotic resistance and genetic diversity of Shigella sonnei isolated from patients with diarrhoea between 1999 and 2003 in Bangladesh. J Med Microbiol 55:1257–1263. doi:10.1099/jmm.0.46641-0 16914657

[15] Khan AI, Rashid MM, Islam MT, Afrad MH, Salimuzzaman M, Hegde ST, Zion MMI, Khan ZH, Shirin T, Habib ZH, Khan IA, Begum YA, Azman AS, Rahman M, Clemens JD, Flora MS, Qadri F. 2020. Epidemiology of cholera in Bangladesh: findings from nationwide hospital-based surveillance, 2014-2018. Clin Infect Dis 71:1635–1642. doi:10.1093/cid/ciz1075 31891368

[16] CLSI G. 2018. Performance standards for antimicrobial susceptibility testing, 29th edition

[17] Khan S, Singh P, Ansari M, Asthana A. 2014. Isolation of Shigella species and their resistance patterns to a panel of fifteen antibiotics in mid and far western region of Nepal. Asian Pac J Trop Dis 4:30–34. doi:10.1016/S2222-1808(14)60309-1

[18] Taneja N. 2007. Changing epidemiology of shigellosis and emergence of ciprofloxacin-resistant Shigellae in India. J Clin Microbiol 45:678–679. doi:10.1128/JCM.02247-06 17122011 PMC1829036

[19] Livio S, Strockbine NA, Panchalingam S, Tennant SM, Barry EM, Marohn ME, Antonio M, Hossain A, Mandomando I, Ochieng JB, et al.. 2014. Shigella isolates from the global enteric multicenter study inform vaccine development. Clin Infect Dis 59:933–941. doi:10.1093/cid/ciu468 24958238 PMC4166982

[20] Thompson CN, Duy PT, Baker S. 2015. The rising dominance of Shigella sonnei: an intercontinental shift in the etiology of bacillary dysentery. PLoS Negl Trop Dis 9:e0003708. doi:10.1371/journal.pntd.0003708 26068698 PMC4466244

[21] Das SK, Ahmed S, Ferdous F, Farzana FD, Chisti MJ, Leung DT, Malek MA, Talukder KA, Bardhan PK, Salam MA, Faruque ASG, Raqib R. 2013. Changing emergence of Shigella sero-groups in Bangladesh: observation from four different diarrheal disease hospitals. PLoS ONE 8:e62029. doi:10.1371/journal.pone.0062029 23658619 PMC3639224

[22] Nandy S, Mitra U, Rajendran K, Dutta P, Dutta S. 2010. Subtype prevalence, plasmid profiles and growing fluoroquinolone resistance in Shigella from Kolkata, India (2001-2007): a hospital-based study. Trop Med Int Health 15:1499–1507. doi:10.1111/j.1365-3156.2010.02656.x 20955371

[23] Zafar A, Hasan R, Nizami SQ, von Seidlein L, Soofi S, Ahsan T, Chandio S, Habib A, Bhutto N, Siddiqui FJ, Rizvi A, Clemens JD, Bhutta ZA. 2009. Frequency of isolation of various subtypes and antimicrobial resistance of Shigella from urban slums of Karachi, Pakistan. Int J Infect Dis 13:668–672. doi:10.1016/j.ijid.2008.10.005 19135399

[24] Vinh H, Nhu NTK, Nga TVT, Duy PT, Campbell JI, Hoang NVM, Boni MF, My PVT, Parry C, Nga TTT, Van Minh P, Thuy CT, Diep TS, Phuong LT, Chinh MT, Loan HT, Tham NTH, Lanh MN, Mong BL, Anh VTC, Bay PVB, Chau NVV, Farrar J, Baker S. 2009. A changing picture of shigellosis in southern Vietnam: shifting species dominance, antimicrobial susceptibility and clinical presentation. BMC Infect Dis 9:204. doi:10.1186/1471-2334-9-204 20003464 PMC2803792

[25] Bangtrakulnonth A, Vieira AR, Lo Fo Wong DMA, Pornreongwong S, Pulsrikarn C, Sawanpanyalert P, Hendriksen RS, Aarestrup FM. 2008. Shigella from humans in Thailand during 1993 to 2006: spatial-time trends in species and serotype distribution. Foodborne Pathog Dis 5:773–784. doi:10.1089/fpd.2008.0109 19086804

[26] Ghosh AR, Sugunan AP, Sehgal SC, Bharadwaj AP. 2003. Emergence of nalidixic acid-resistant Shigella sonnei in acute-diarrhea patients on Andaman and Nicobar Islands, India. Antimicrob Agents Chemother 47:1483. doi:10.1128/AAC.47.4.1483.2003 12654701 PMC152529

[27] Zhang W, Luo Y, Li J, Lin L, Ma Y, Hu C, Jin S, Ran L, Cui S. 2011. Wide dissemination of multidrug-resistant Shigella isolates in China. J Antimicrob Chemother 66:2527–2535. doi:10.1093/jac/dkr341 21859815

[28] Rahman M, Haque AF, Deeba IM, Ahmed D, Zahidi T, Rimu AH, Akter M, Akter F, Talukder KA. 2017. Emergence of extensively drug-resistant Shigella sonnei in Bangladesh. Immunol Infect Dis 5:1–9. doi:10.13189/iid.2017.050101

[29] Azmi IJ, Khajanchi BK, Akter F, Hasan TN, Shahnaij M, Akter M, Banik A, Sultana H, Hossain MA, Ahmed MK, Faruque SM, Talukder KA. 2014. Fluoroquinolone resistance mechanisms of Shigella flexneri isolated in Bangladesh. PLoS ONE 9:e102533. doi:10.1371/journal.pone.0102533 25028972 PMC4100904

[30] Kuo C-Y, Su L-H, Perera J, Carlos C, Tan BH, Kumarasinghe G, So T, Van PH, Chongthaleong A, Song J-H, Chiu C-H. 2008. Antimicrobial susceptibility of Shigella isolates in eight Asian countries, 2001-2004. J Microbiol Immunol Infect 41:107–111. 18473096

[31] von Seidlein L, Kim DR, Ali M, Lee H, Wang X, Thiem VD, Canh DG, Chaicumpa W, Agtini MD, Hossain A, Bhutta ZA, Mason C, Sethabutr O, Talukder K, Nair GB, Deen JL, Kotloff K, Clemens J. 2006. A multicentre study of Shigella diarrhoea in six Asian countries: disease burden, clinical manifestations, and microbiology. PLoS Med 3:e353. doi:10.1371/journal.pmed.0030353 16968124 PMC1564174

[32] Platts-Mills JA, Liu J, Rogawski ET, Kabir F, Lertsethtakarn P, Siguas M, Khan SS, Praharaj I, Murei A, Nshama R, et al.. 2018. Use of quantitative molecular diagnostic methods to assess the aetiology, burden, and clinical characteristics of diarrhoea in children in low-resource settings: a reanalysis of the MAL-ED cohort study. Lancet Glob Health 6:e1309–e1318. doi:10.1016/S2214-109X(18)30349-8 30287127 PMC6227251

[33] Rogawski ET, Liu J, Platts-Mills JA, Kabir F, Lertsethtakarn P, Siguas M, Khan SS, Praharaj I, Murei A, Nshama R, et al.. 2018. Use of quantitative molecular diagnostic methods to investigate the effect of enteropathogen infections on linear growth in children in low-resource settings: longitudinal analysis of results from the MAL-ED cohort study. Lancet Glob Health 6:e1319–e1328. doi:10.1016/S2214-109X(18)30351-6 30287125 PMC6227248

[34] Raqib R, Sarker P, Zaman K, Alam NH, Wierzba TF, Maier N, Talukder K, Baqui AH, Suvarnapunya AE, Qadri F, Walker RI, Fix A, Venkatesan MM. 2019. A phase I trial of WRSS1, a Shigella sonnei live oral vaccine in Bangladeshi adults and children. Hum Vaccin Immunother 15:1326–1337. doi:10.1080/21645515.2019.1575165 30794051 PMC6663145

